# Melatonin is a potential oncostatic agent to inhibit HepG2 cell proliferation through multiple pathways

**DOI:** 10.1016/j.heliyon.2022.e08837

**Published:** 2022-01-31

**Authors:** Omar A. Ammar, Mohamed A. El-Missiry, Azza I. Othman, Maggie E. Amer

**Affiliations:** a Basic Science Department, Delta University for Science and Technology, Gamasa, Egypt; b Zoology Department, Faculty of Science, Mansoura University, Egypt

**Keywords:** Chemotherapy, Liver cancer, Apoptosis, Angiogenesis, Cell cycle, Melatonin

## Abstract

**Context:**

Chemotherapy is a cornerstone in the treatment of hepatocellular carcinoma (HCC). Melatonin is a pineal hormone that targets various cancers, however, its antitumor pathways are still not fully elucidated.

**Objective:**

This study investigated melatonin's antitumor molecular mechanisms to inhibit the proliferation of HepG2 cells.

**Materials and methods:**

HepG2 Cells were classified into cells without treatment as a control group and cells treated with melatonin (5.4 mmol/L) for 48 h. Proliferating cell nuclear antigen (PCNA) and marker of proliferation Ki-67 were estimated using immunohistochemical analysis. Apoptosis and cell cycle were evaluated using flow cytometric analysis. Apoptotic markers were detected using RT-qPCR assay. Antioxidants and oxidative stress biomarkers were performed using a colorimetric assay.

**Results:**

Melatonin produced a remarkable steady decrease in the viability of HepG2 cells at a concentration range between 5-20 mmol/L. Melatonin suppressed cell proliferation in the G2/M phase of the cell cycle (34.97 ± 0.92%) and induced apoptosis (12.43 ± 0.73%) through up-regulating p21 and p53 that was confirmed by the reduction of PCNA and Ki-67 expressions. Additionally, melatonin repressed angiogenesis evidenced by the down-regulation of angiopoietin-2, vascular endothelial growth factor receptor-2 expressions (0.42-fold change), and the level of CD133. Moreover, melatonin augmented the oxidative stress manifested by a marked increase of 4-hydroxynonenal levels with a reduction of glutathione content and superoxide dismutase activity.

**Discussion and conclusion:**

Melatonin inhibits proliferation and angiogenesis and induced apoptosis and oxidative stress in HepG2 cells. These results indicate the oncostatic effectiveness of melatonin on liver cancer.

## 1 Introduction

Hepatocellular carcinoma (HCC) is life-threatening cancer and deserves considerable research for finding successful chemotherapy (Yang et al., 2019). Chemotherapy either alone or with other modalities is the main intervention option for HCC. Unfortunately, the cytotoxicity and harmful side effects of drugs used for HCC are limiting factors in chemotherapy of patients. Thus, searching for effective and safe antitumor drugs is an essential issue. The most common treatment choice for early-stage (A) and intermediate-stage (B) cancer, respectively, is surgery and chemotherapy (Llovet et al., 2016).

It is reported that HCC cells can resist conventional chemotherapeutic drugs (Jin et al., 2015). Moreover, in malignant cells, the pathways that regulate the responses of pro-oncogenes and tumor suppressor genes are not well described in liver cancer cells. The main mechanism of cancer growth and progression is avoiding apoptosis and accelerating cell division (Lin et al., 2017). Therefore, selective apoptosis inductors and cell cycle suppressors have emerged as a promising strategy for developing a specific drug for cancer treatment (Mortezaee et al., 2019). Angiogenesis is an important process for cancer growth and progression and blocking this process is an effective approach in the treatment of cancers.

The most widely used model of liver cancer is the human hepatocellular carcinoma cell line (HepG2) because it is a permanent cell line originally derived from a well-differentiated patient with hepatocellular carcinoma. These cells are widely used in research on liver cell metabolism and evaluating several drug actions (Gerets et al., 2012).

Melatonin is a hormone mainly synthesized and released by the pineal gland. It is characterized by a wide range of actions (Sartorelli et al., 2021). Besides its physiological role in regulating circadian rhythms and reproductive system, its activity includes antitumor, immunomodulatory, and antioxidant actions as well as free radical scavenging activity were documented (Voiculescu et al., 2014; Karaaslan and Suzen 2015; Reiter et al., 2021). Despite its well-known antioxidant function to protect normal cells from cytotoxicity and apoptosis, melatonin showed prooxidant effects (Gurer-Orhan and Suzen 2015) and induce apoptosis in several cancer cells (Mortezaee et al., 2019). It is reported that melatonin can exert a prooxidant effect through multiple mechanisms including depletion of glutathione levels in a human myeloid leukemia cell line (U937) (Albertini et al., 2006) and HepG2 cells (Osseni et al., 2000). Additionally, melatonin induces apoptosis via disruption of mitochondrial function leading to the liberation of cytochrome c and activation of caspases (Pourhanifeh et al., 2019). Melatonin was reported to activate the extrinsic apoptotic pathway by increasing the expression of Fas ligands (Wölfler et al., 2001). Many epidemiological studies support its anticancer activity (Li et al., 2017; Nooshinfar et al., 2017; Amin et al., 2019) in several cancer cell lines. Melatonin has been found to suppress various carcinomas *in vivo* and *in vitro*, such as lymphoma (Sánchez-Hidalgo et al., 2012), gastric cancer (Zhang et al., 2013; Wang et al., 2016b), liver (Carbajo-Pescador et al., 2011; Ordoñez et al., 2014), breast (Alvarez-Garcia et al., 2013; Proietti et al., 2014; Amin et al., 2019; Chuffa et al., 2020), oral (Cutando et al., 2014; Goncalves Ndo et al., 2014), glioma (Gu et al., 2017; Maitra et al., 2019), ovary (Ferreira et al., 2014; Chuffa et al., 2017), and prostate cancer (Rodriguez-Garcia et al., 2013; Paroni et al., 2014). Melatonin was reported to have antiangiogenic effects by modulating vascular endothelial growth factor (VEGF) and hypoxia-inducible factors in several culture cell lines (Dai et al., 2008; Park et al., 2010).

The anticancer effect of melatonin on liver cancer is an important issue however its oncostatic effectiveness is unclear and therefore, deserves additional characterization of its anticancer mechanisms. The current study aimed to investigate the molecular antitumor mechanism of melatonin in the HepG2 cell line of human hepatoma. Biomarkers linked to cell apoptosis, proliferation, angiogenesis, and oxidative stress were used to examine the possible molecular anticancer responses induced by melatonin.

## 2 Materials and methods

### 2.1 Cell culture and treatment

The Holding Company for Biological Products & Vaccines (VACSERA, Egypt) provided human hepatocellular carcinoma (HepG2) cells. At 37 °C and 5% CO2, HepG2 cells were cultured in DMEM supplemented with 10% (v/v) fetal bovine serum, 2 mmol/L glutamine, 100 U/mL penicillin, and 100 μg/mL streptomycin. All used media and reagents were purchased from (Lonza Bioproducts, Belgium). Experimental groups were classified into two groups. The first group is defined as HepG2 cells without treatment. The second group is defined as HepG2 cells treated with melatonin IC_50_ dose for 48 h.

### 2.2 MTT cytotoxicity assay and estimation of IC_50_

Melatonin's effect on cell viability was determined using the MTT assay. HepG2 cells were seeded at 7×10^3^ cells/well in a 96-well plate for 48 h at 37 °C in 95% air and 5% CO_2_ atmosphere, with 95% humidity (Kumar et al., 2018). Melatonin dissolved in dimethyl sulphoxide (DMSO). The final concentrations of melatonin in the wells were ranged between 0 to 20000 μmol/L for 48 h. After 48 h of treatment, the medium was removed from each well and 50 μL of 3-(4,5-dimethylthiazol-2-yl)-2,5-diphenyltetrazolium bromide (MTT) (5 mg/mL; Sigma, St. Louis, CA, USA) was added. The plates were gently shaken before being incubated at 37 °C for an additional 4 h in the dark. After stopping the reaction with 150 μL DMSO (Sigma), the absorbance of the samples at 570 nm was measured using a microplate reader (SunRise, Tecan, USA).

### 2.3 Immunohistochemical analysis of proliferating cell nuclear antigen (PCNA) and marker of proliferation (Ki-67)

HepG2 cell incubated with PCNA antibody (Santa Cruz biotechnology, sc-56, 1:300 dilution) and primary monoclonal anti-mouse antibody for Ki-67 protein (Santa Cruz Biotechnology, code: sc-23900, dilution 1:200) overnight at 4 °C. After washing, the cells were incubated with HRP-conjugated secondary antibodies for 1 h at room temperature. After 3, 3′-diaminobenzidine (DAB) incubation, cells were counterstained with hematoxylin and observed under an optical microscope in a population of 10^3^ cells sampled at about × 200 magnification. Each brown stained nucleus, regardless of intensity, was considered positive and counted to quantify HepG2 cell proliferation.

### 2.4 Cell cycle analysis

HepG2 cells were collected and fixed with 75% ice-cold ethanol before being stored at -20 °C for 1 h after being treated with an IC_50_ dose of melatonin. Centrifuged cells were harvested, washed twice with ice-cold PBS, and incubated with PI for 20 min at 4 °C. A cell cycle assay was used to assess the cell cycle (Propidium Iodide Flow Cytometry Kit [ab139418], FACSCalibur; BD Biosciences, CA, USA). FlowJo 7.6 software (TreeStar, San Carlos, CA, USA) was used to perform statistical analysis on the cell fractions in sub-G0, G0/G1, S, and G2/M phases. Each concentration was measured four times, and the findings of six separate trials are shown.

### 2.5 Apoptosis detection

HepG2 cells were subjected to apoptosis determination using BD FACSCalibur Flow Cytometer (BD Biosciences, CA, USA) and Annexin V-FITC Apoptosis Detection Kit (BioVision, USA) Catalog #: K201.

### 2.6 Real-time quantitative PCR (RT-qPCR) assay

The control and treated groups' total RNA were then isolated. RNeasy Mini Kits (QIAGEN, Venlo, Netherlands) were used to extract RNA according to the manufacturer's protocol. On a Rotor-Gene Q real-time PCR cycler (QIAGEN, Venlo, Netherlands), RT-qPCR was carried out with SYBR® Green dye (iScriptTM One-Step RT-PCR Kit, Bio-Rad, California, United States). The forward and reverse primers for P21, P53, Bcl-2 associated X (Bax), B-cell lymphoma 2 (Bcl-2), cysteine-aspartic acid protease-3 (caspase-3), and vascular endothelial growth factor receptor 2 (VEGFR2) are mentioned in Table 1. Quantitative analysis was conducted during the exponential amplification process by estimating the values of the threshold cycle (CT). The discrepancy between the CT values of the P21, P53, Bax, Bcl-2, caspase-3, VEGFR2, and the CT value of the glyceraldehyde 3-phosphate dehydrogenase (GADPH) housekeeping gene was used to detect ΔCT. Finally, the comparative Ct procedure for relative quantification (2^−ΔΔCt^) was used to assess fold changes in treated HepG2 cells compared to untreated HepG2 cells (Livak and Schmittgen 2001).

**Table 1:** Primer sequences used in quantitative real-time PCR experiment.

Gene	Forward	Reverse
GAPDH	5′-GAAGGTGAAGGTCGGAGTCA-3′	5′-TTGAGGTCAATGAAGGGGTC-3′
P53	5′-CCCCTCCTGGCCCCTGTCATCTTC-3′	5′-GCAGCGCCTCACAACCTCCGTCAT-3′
Bax	5′-GTT TCA TCC AGG ATC GAG CAG-3′	5′-CAT CTT CTT CCA GAT GGT GA-3′
Bcl-2	5′-CCTGTG GAT GAC TGA GTA CC-3′	5′-GAGACA GCC AGG AGA AAT CA-3′
Caspase-3	5′-TTC ATT ATT CAG GCC TGC CGA GG-3′	5′-TTC TGA CAG GCC ATG TCA TCC TCA-3′
p21	5′- GTCACTGTCTTGTACCCTTGTG-3′	5′-CGGCGTTTGGAGTGGTAGAAA-3′
VEGFR2	5′-CAGTGGGCTGATGACCAAGA-3′	5′-GGGTGGGACATACACAACCA-3′

### 2.7 Biochemical determinations

#### 2.7.1 Estimation of CD133

CD133 cancer stem cell biomarker was detected using a CD133 (Human) ELISA Kit (Biovision, Catalog #: K4192). Cell culture supernatant centrifuged for 20 min to remove insoluble impurity and cell debris at 1000 *g* at 2–8 °C. The clear supernatant was collected to carry out the assay, and then the result was read at 450 nm within 20 min using a LUMIstar OPTIMA (Thermo Fisher).

#### 2.7.2 Estimation of angiogenesis indicator

The antiangiogenic activity was evaluated by measuring the angiopoietin-2 (Ang-2) protein level. Cell culture supernatant was used to measure Ang-2 protein level at 450 nm within 20 min by ELISA (R&D, #DANG20) according to the manufacturer's instructions.

#### 2.7.3 Oxidative stress and antioxidants levels

Oxidative stress was determined by estimating the 4-hydroxynonenal (4HNE) level. HepG2 cell incubated with anti-4-HNE antibody at room temperature for 1 h on an orbital shaker. After washing, the cells were incubated with secondary antibody-HRP conjugate for 1 h at room temperature. After stopping the reaction with 100 μL of 2 M sulfuric acid stop solution (Abcam # ab238538, Cambridge, MA, USA), the absorbance of the samples at 450 nm was measured using a microplate reader. Glutathione content in HpG2 cells was estimated using the following instruction manual DetectX® glutathione fluorescent detection kit (Arbor Assays, Ann Arbor, MI, USA). The detection method uses ThioStar glutathione detection reagent, which is a fluorescent reagent that binds to glutathione (GSH). All measurements were done in triplicate using a 96 microplate with LUOstar OPTIMA plate reader (BMG Labtechnologies) at 510 nm with excitation at 370-410 nm. The antioxidant activity was detected by superoxide dismutase (SOD). A superoxide dismutase assay kit (#706002, Cayman Chemicals Inc., Ann Arbor, Michigan, USA) was used to assess SOD activity. After adding the radical detector, the reaction was initiated by xanthine oxidase then the absorbance was measured at 440-460 nm using a plate reader.

### 2.8 Statistical analysis

The mean and standard deviation were used to present quantitative parametric data, whilst the median and range (minimum-maximum) were used to present non-parametric data. For quantitative parametric analysis, the Student's t-test was used, while for quantitative non-parametric comparisons, the Mann Whitney test was used. The data was analyzed using Version 23 of the Statistical Package (SPSS, Inc., Chicago, IL, USA). Statistical significance is defined as a *P* value of less than 0.05.

## 3 Results

### 3.1 Melatonin is cytotoxic to HepG2 cells

**Figure 1:**
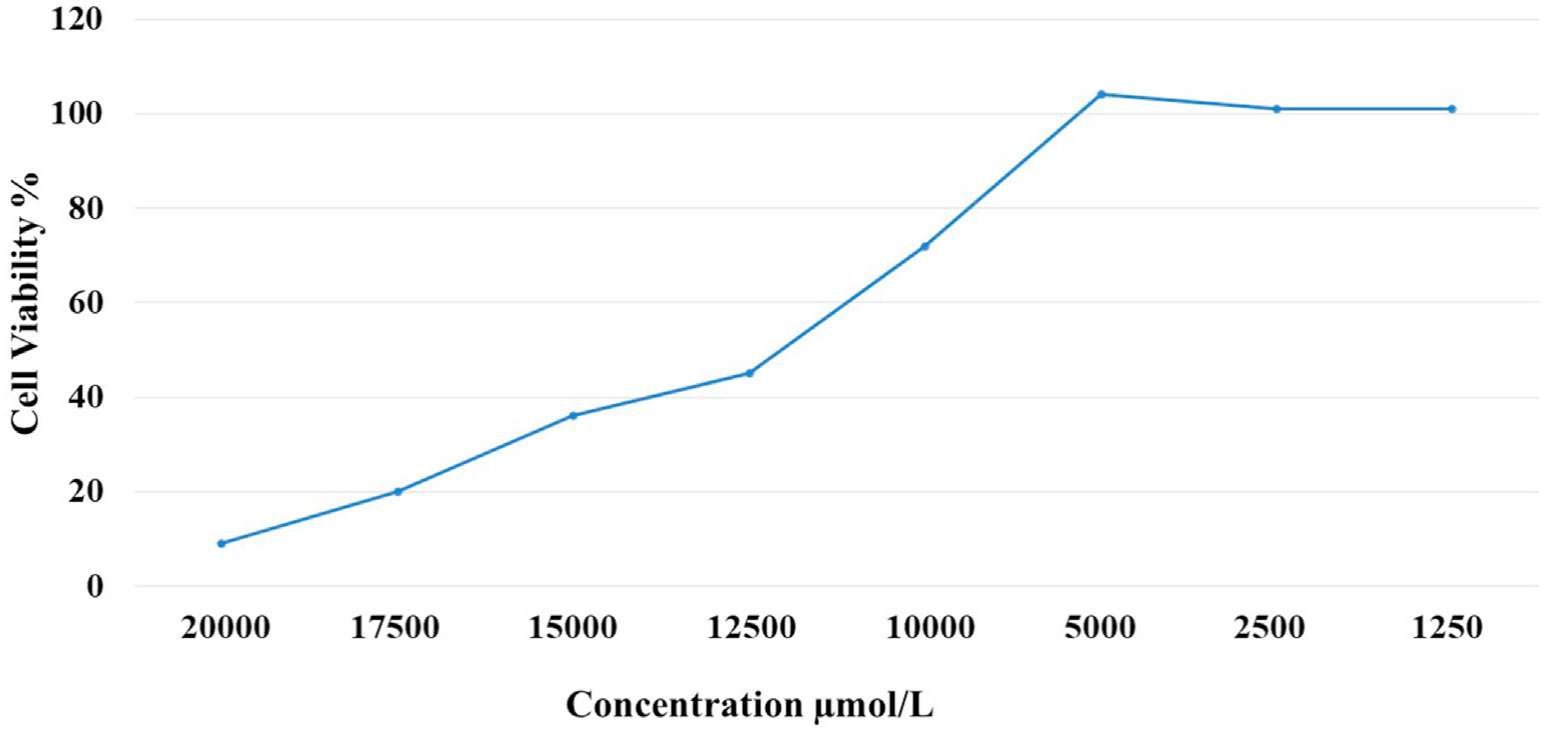
Effect of melatonin on viability HepG2 cells. HepG2 cells was incubated with different concentrations of melatonin for 48 h. The viability was estimated as percent of MTT uptake.

MTT assay demonstrated dose-dependent inhibition of cell growth after exposure to melatonin (Figure 1). The mean concentration that inhibited 50% of cell growth (IC_50_) was detected by an average of the individual results at 48 h from the three repeated experiments. The melatonin IC_50_ dose was 5.4 mmol/L.

### 3.2 Melatonin decreased Ki-67 and PCNA

**Figure 2:**
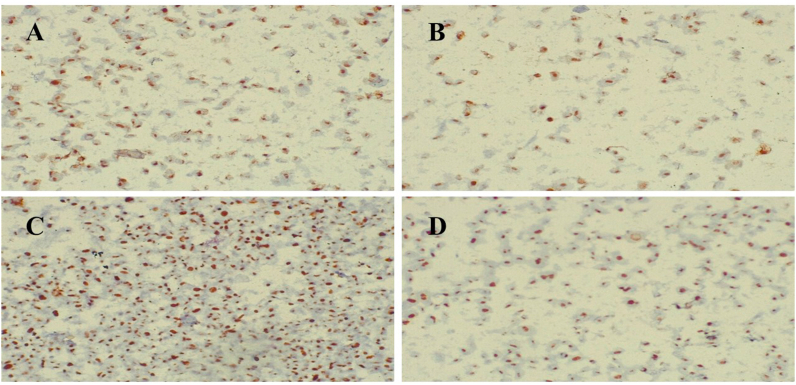
Effect of melatonin treatment on expression levels of cellular PCNA and Ki-67 in Hepg2 hepatocarcinoma cells. Immunohistochemical staining for PCNA and Ki-67 in HepG2 cells. (A), PCNA expression in untreated HepG2 cells and (B), PCNA expression was donregulated in melatonin-treated HepG2 cells. (C), Ki-67 expression in untreated HepG2 cells and (D) Ki-67 expression was decreased in melatonin-treated HepG2 cells. (Original magnification×200).

The expression and localization of PCNA and Ki-67 in both melatonin-treated HepG2 cells and control were examined immunohistochemically in three repeated experiments (Figure 2). PCNA and Ki-67 were expressed in 53.7 ± 2.24% and 77.4 ± 2.2% respectively in control. The percentages of PCNA and Ki-67 expression were significantly decreased (*P* < 0.001) by 24.7 ± 1.07% and 39.4 ± 1.7% respectively after treatment with melatonin. These findings indicate that melatonin caused a significant decrease in the proliferation of HepG2 cells.

### 3.3 Melatonin induced cell cycle arrest in HepG2 cells

**Figure 3:**
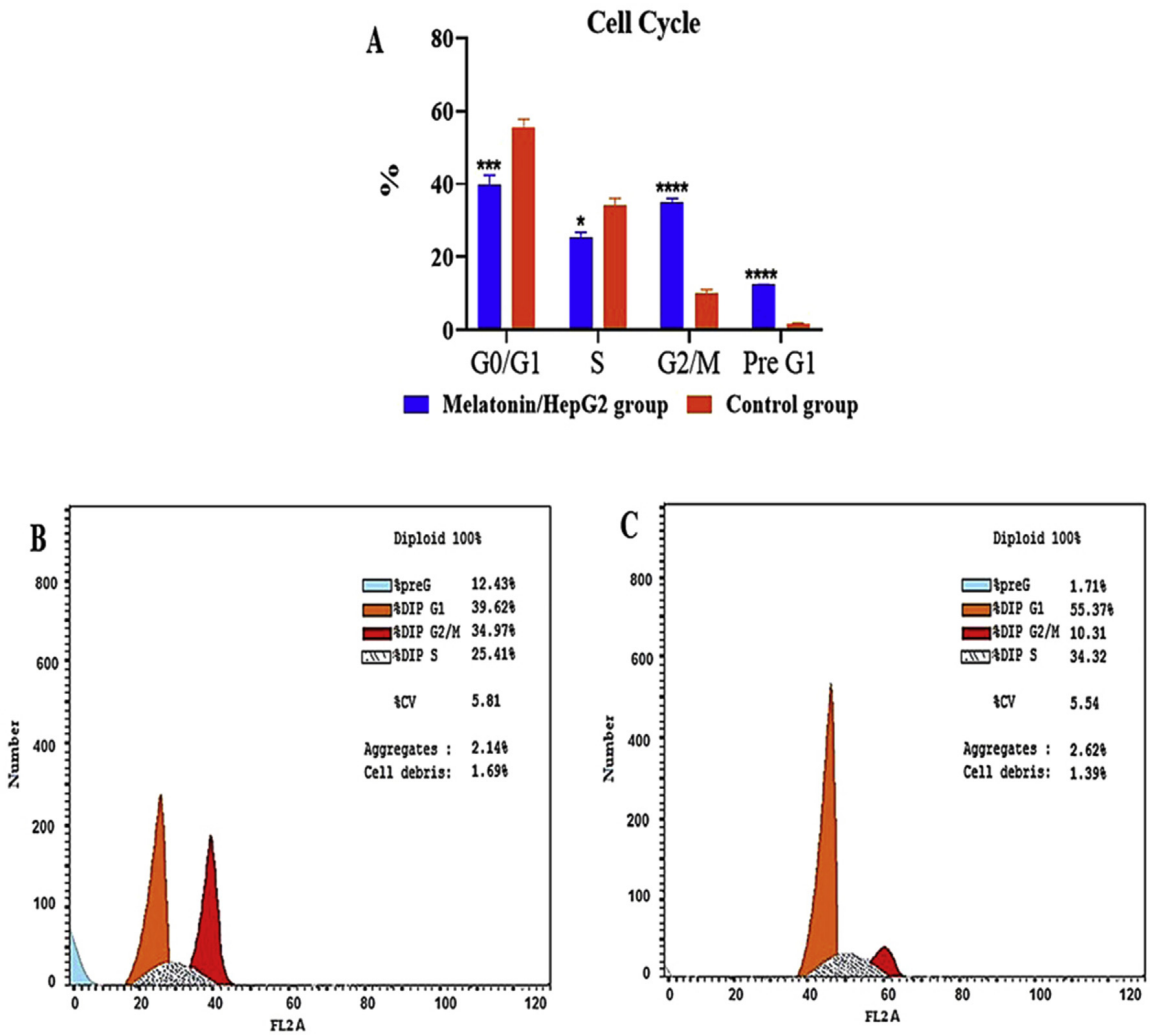
Effect of melatonin treatment on cell cycle in HepG2 hepatocarcinoma cells. (A) Flow cytometry analysis of HepG2 hepatocarcinoma cells. (B and C) representative charte of flow cytometer for melatonin-treated HepG2 cells and untreated cells respectively. Each panel represents the relative number of cells according to the DNA content in each cell.

The HepG2 cell cycle distribution was detected by flow cytometry (Figure 3). The current study demonstrated that the percentage of the population of HepG2 cells in both G0/G1 and S-phage in melatonin-treated cells was significantly (*P* < 0.001) lower than untreated cells. On the other hand, the cell population in G2/M was significantly (*P* < 0.001) higher in melatonin-treated cells than untreated cells. These findings indicate cell cycle arrest of HepG2 cells treated with melatonin at the G2/M phase.

### 3.4 Melatonin induced apoptosis in HepG2 cells

**Figure 4:**
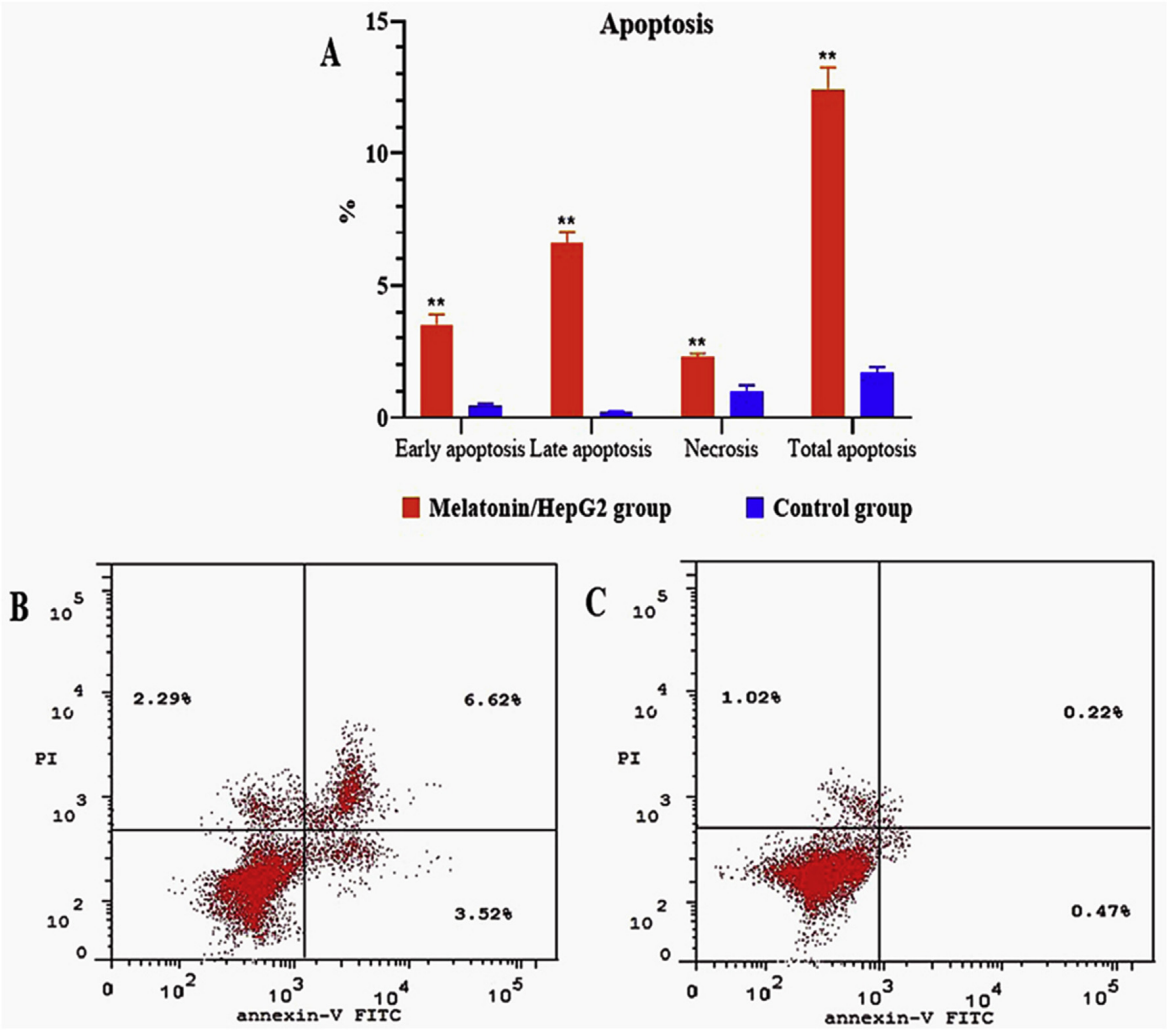
Effect of melatonin on apoptosis measured by flow cytometry in HepG2 hepatocellular carcinoma cells after 48 h of treatement with melatonin (A).(B and C) Flow cytometric histograms of apoptosis in HepG2 cells with and without melatonin treatment respectively.

Both the untreated control and the melatonin treated HepG2 cells in three repeated experiments showed the percentages of the early apoptotic cells as ranging from 0.47 ± 0.05% to 3.52 ± 0.35% (*P* < 0.001); the percentages of the late apoptotic cells ranging from 0.22 ± 0.02% to 6.62 ± 0.35% (*P* < 0.001); and the percentages of necrosis as ranging from 1.02 ± 0.19% to 2.29 ± 0.12% (*P* < 0.001) (Figure 4).

### 3.5 Melatonin enhanced apoptotic regulating proteins in HepG2 cells

Gene expression of apoptotic proteins was evaluated using qRT-PCR and displayed in Table 2. The data showed that the expression levels of p21, P53, Bax, and caspase-3 were significantly upregulated in cells treated with melatonin more than untreated cells. This was paralleled with a significant (*P* < 0.001) decrease in the expression levels of antiapoptotic protein Bcl-2 in melatonin-treated cells.

**Table 2:** Statistical analysis of P21, P53, Bax, caspase-3, and Bcl-2 gene expression in melatonin-treated HepG2 cells and control using qRT-PCR analysis.

Gene Expression	Melatonin/HepG2 group	Control group	*P*
P21	2.3 (2.09–2.51)	1.00 (1.00–1.00)	<0.001∗
P53	13.05 (12.36–13.74)	1.00 (1.00–1.00)	<0.001∗
Bax	7.74 (7.36–8.12)	1.00 (1.00–1.00)	<0.001∗
Caspase-3	4.94 (4.68–5.2)	1.00 (1.00–1.00)	<0.001∗
Bcl-2	0.66 (0.62–0.7)	1.00 (1.00–1.00)	<0.001∗

*P*: Probability

∗: significance <0.05.

Mann whitney for data expressed as median (range).

### 3.6 Melatonin increased oxidative stress in HepG2 cells

The prooxidant effect of melatonin on HepG2 cells was evaluated and displayed in Table 3. Incubation of HepG2 cells with melatonin showed a significant (*P* < 0.001) increase of lipid peroxidation product, 4HNE. Furthermore, The SOD activity and GSH content were significantly decreased (*P* < 0.001) compared with the untreated HepG2 cells.

**Table 3:** Statistical analysis of lipid peroxidation biomarkers (4HNE) and antioxidants (SOD activity and GSH content) in melatonin-treated HepG2 cells and control using ELISA assay.

Biomarker	Melatonin/HepG2 group	Control group	*P*
4-HNE (μg/1×10^6^ cells)	13.71 ± 0.51	5.37 ± 0.44	<0.001∗
SOD (U/mg protein)	1.84 ± 0.04	2.85 ± 0.06	<0.001∗
GSH (nmol/mg protein)	1.87 ± 0.01	3.74 ± 0.14	<0.001∗

*P*: Probability

∗: significance <0.05.

Student's t-test for data expressed as mean ± SEM.

### 3.7 Melatonin reduced angiogenesis and CD133 in HepG2 cells

**Figure 5:**
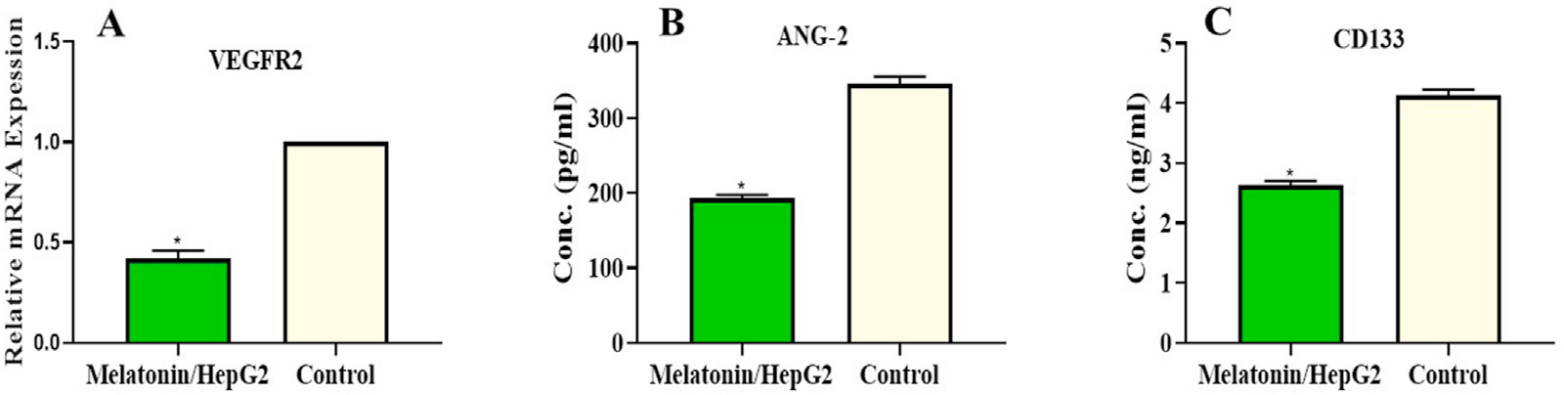
Effect of melatonin on angiogenesis (VEGFR2 & ANG-2) and cancer stem cell CD133 biomarkers in melatonin-treated HepG2 cells and control using ELISA assay. (A) VEGFR2 gene expression, (B) ANG-2 protein level and (C) CD133 cancer stem cell level.

In melatonin-treated HepG2 cells, the gene expression of VEGFR2 was significantly down-regulated compared with the untreated cells. In addition, the expression level of ANG-2 was significantly decreased in HepG2 cells treated with melatonin. Moreover, a significantly low level of CD133 was demonstrated in the cells treated with melatonin compared with control cells. These results were detected through three repeated experiments (Figure 5).

## 4 Discussion

Hepatocellular carcinoma is the most common liver cancer and still, there is a lack of effective therapy with minimal side effects (Neamatallah et al., 2014; Ho et al., 2016). Because melatonin is a well-known, safe pharmaceutical compound with a remarkable anticancer potential *in vivo* and *in vitro* (El-Missiry & Abd El-Aziz 2000), it is anticipated that it could be an effective agent in the treatment of liver cancer. The current study reveals that melatonin has multiple molecular pathways and targets to exert its antitumor impact on the growth and proliferation of HepG2 cells. Melatonin's rhythm is frequently disturbed in cancer patients, and its amount is also decreased. The alterations in melatonin impair its antioxidant and circadian regulating activities on cells and tissues, rendering them more susceptible to mutations and cancer development (Sartorelli et al., 2021).

The current data showed a remarkable decrease in the viability of HepG2 cells after melatonin treatment evidenced by a decrease in Ki-67 and PCNA. PCNA is an essential protein for human cells to synthesize DNA thus indicating the proliferation activity. PCNA is an indispensable regulator of DNA replication, repair, cell cycle control, and apoptosis (Shemesh et al., 2018). Ki-67 is a nuclear antigen whose high expression, which is produced from cell proliferation, is closely associated with all phases of mitosis except G0 (Li et al., 2018). Thus, the overexpression of PCNA and Ki-67 is correlated with cancer progression, and targeting these nuclear antigens by melatonin has a remarkable inhibitory impact on HepG2 cell proliferation. Our findings support a previous study that found melatonin reduced cell viability and inhibited the proliferation of HepG2 cells (Li et al., 2017). In several tumor types, such as breast cancer, lymphoma, and neuroendocrine tumors, Ki67 is also a good prognostic biomarker (Takkem et al., 2018). Therefore, the current study confirms the inhibition of cell growth and proliferation of HepG2 cells by melatonin and signifying its chemotherapeutic effectiveness on liver cancer.

Typically, the cell cycle is decontrolled in cancer cells leading to increased cell proliferation (Otto and Sicinski 2017). Consequently, suppression of the cell cycle is a suitable approach for cancer treatment as well as diseases characterized by increased proliferation (Mills et al., 2018). We demonstrated that treatment of HepG2 cells with melatonin significantly induced G2/M phase arrest confirming earlier study (Bennukul et al., 2014). When DNA is damaged, the G2 checkpoint prevents cells from starting mitosis and ensures that each daughter cell receives error-free copies of the genome. The Cdk1/Cyclin B1 complex regulates protein phosphorylation and dephosphorylation to control cell cycle progression from G2 to M phase. Furthermore, coordinated actin remodeling can ensure proper G2/M checkpoint arrest and is necessary for mitotic entrance (Wang et al., 2016a). The current results agree with the suggestion that upregulation of p21 by melatonin has a strong inhibitory effect on the cell cycle. Early studies showed that melatonin significantly increased p21 which is the main cancer-inhibitory mechanism (Proietti et al., 2014; Zhang et al., 2019). Immunohistochemical studies demonstrated reduced expression of Ki-67 and PCNA with melatonin treatment in HepG2 cells, confirming cell cycle arrest HepG2 proliferation. Supporting a previous study (Sun et al., 2017), the decrease in the expression of Ki67 protein is also correlated with the upregulation of p21 expression in many cell lines, such as breast cancer cells of MCF7. Thus, melatonin-induced upregulation of p21 is considered to be an important cancer-inhibitory mechanism.

CD133 is a surface glycoprotein, which has been considered a potential target for cancer treatment, and the suppression of CD133 expression is considered to be a plausible approach for the progression of cancer (Glumac and LeBeau 2018). The present study demonstrated a significant decrease in CD133 levels after incubation of HepG2 cells with melatonin. However, the precise mechanism explaining the decrease of CD133 expression by melatonin is still unclear. It was reported that Knockdown of CD133 reduced G0/G1 phase cells and increased cellular apoptosis via modulation of Bcl-2 and Bax (Lan et al., 2013). Thus, we anticipate that the oncostatic effect of melatonin might be attributed to silencing effects on CD133 expression on HepG2 cells.

Avoiding apoptosis in HCC is an important factor involved in liver cancer initiation and its progression and is highly correlated with a low response to conventional chemotherapy (Fan et al., 2013). Melatonin enhanced cellular apoptosis by up-regulating P21, P53, and the pro-apoptotic protein Bax production and down-regulating the antiapoptotic protein Bcl-2 in HepG2 cells confirming another study (Talib 2018). We also observed that treatment with melatonin caused significant activation of caspase-3, confirming the apoptosis-inducing effect of melatonin on HepG2 cells. These findings confirm previous studies that melatonin inhibits apoptosis resistance and activate both extrinsic and intrinsic pathways of apoptosis in HCC (Martín-Renedo et al., 2008; Fan et al., 2013; Mortezaee et al., 2019). Thus, it is possible to suggest that melatonin could be a promising oncostatic agent for liver cancer acting through a caspase-dependent pathway of apoptosis. In this respect, melatonin has previously been shown to have antiproliferative and proapoptotic impacts in an *in vitro* model of HCC (Carbajo-Pescador et al., 2011; Colombo et al., 2016) confirming that melatonin provided a good platform for cancer chemotherapy.

Cancer proliferation and survival essentially depend on the development of new blood vessels (Bergers and Benjamin 2003). Angiogenesis is the fundamental process in tumor growth and antiangiogenic agents are promising and suitable strategies in cancer therapy (Bareschino et al., 2011). The results of this study revealed inhibitory effects of melatonin on angiogenesis via the inhibition of Ang-2 and VEGFR2 expression in HepG2 cells that is consistent with other recent studies (Goradel et al., 2017; González-González et al., 2020). Melatonin reduced the levels of VEGF, VEGFR2, and HIF-1α, which could be mediated through its receptor MT1 (Zonta et al., 2017). VEGF and VEGFR2 are important elements for the regulation of vascularization and angiogenesis. It is demonstrated *in vitro* study that VEGF increased CD133 expression via activation of VEGFR2 (Liu et al., 2017). The reduced Ang-2 enables VEGF to block angiogenesis (Chae et al., 2010). Thus, it is suggested that melatonin might be considered anti-VEGFR2 that reduces the Ang-2 expression. These findings show the potential of melatonin in functioning as a natural antiangiogenic compound by targeting Ang-2 and VEGFR2. Recent studies reported that melatonin blocks are proangiogenic and potentiate antiangiogenic effects induced by docetaxel and vinorelbine enhancing their antitumor effects in human umbilical vein endothelial cells (González-González et al., 2020). In addition, melatonin reduced the expression of VEGF mRNA and inhibits proliferation, invasion, migration of endothelial cells, and tubular network formation induced by VEGF (Alvarez-Garcia et al., 2013). Similarly, melatonin indirectly inhibits angiogenesis through scavenging ROS, which has an important function in stabilizing hypoxia-inducible factor HIF-α during hypoxia (Fandrey and Genius 2000). Thus, melatonin can exert antiangiogenic actions to inhibit liver cancer proliferation and growth.

Oxidative stress is an important mediator of HCC and a common pathologic feature for a variety of HCC models (Choi et al., 2014). Oxidative stress is induced by a decrease in cellular antioxidant defenses leading to ROS overproduction. Melatonin is a potent antioxidant in normal cells or tissues under oxidative stress and exerts a clear oncostatic effect in many cancer cell types including pancreatic stellate cells (Estaras et al. 2019, 2020; Gonzalez et al., 2020) and pancreatic AR42J cells (Gonzalez et al., 2011; Uguz et al., 2012). Melatonin becomes a prooxidant agent to enhance oxidant stress to cause cell death. The present study showed that melatonin increased lipid peroxidation product (4-HNE) with a significant decrease in GSH contents and SOD activity in HepG2. These findings suggest that melatonin has pro-oxidant properties in HepG2 cells. These findings support earlier studies by (Osseni et al., 2000), who demonstrated that melatonin can have a pro-oxidant effect, depending on its concentration and the duration of incubation in HepG2 hepatocarcinoma cells. It is recently reported that melatonin inhibits the progression of tumors due to its, prooxidant effect in several cancer types including non-small-cell lung cancer (Pourhanifeh et al., 2019), breast cancer (Li et al., 2017; El-Sokkary et al., 2019), and leukemia (Shafabakhsh et al., 2020).

## 5 Conclusion

The present study demonstrated that melatonin inhibits tumor growth and progression of HepG2 cells, through multiple pathways including induction of apoptosis and reduction of angiogenesis and pro-oxidation effect. These pathways act simultaneously to inhibit cancer proliferation and progression. Thus, it is suggested that melatonin can be used as a promising potential therapeutic strategy for liver cancers.

## Declarations

### Author contribution statement

M A El-Missiry; Azza I Othman; Omar A. Ammar; Maggie E Amer: Conceived and designed the experiments; Analyzed and interpreted the data; Wrote the paper.

Omar A. Ammar; Maggie E Amer: Performed the experiments; Contributed reagents, materials, analysis tools or data.

### Funding statement

This research did not receive any specific grant from funding agencies in the public, commercial, or not-for-profit sectors.

### Data availability statement

Data included in article/supplementary material/referenced in article.

### Declaration of interests statement

The authors declare no conflict of interest.

### Additional information

No additional information is available for this paper.
